# Recognition of Highly Diverse Type-1 and -2 Porcine Reproductive and Respiratory Syndrome Viruses (PRRSVs) by T-Lymphocytes Induced in Pigs after Experimental Infection with a Type-2 PRRSV Strain

**DOI:** 10.1371/journal.pone.0165450

**Published:** 2016-10-31

**Authors:** Chungwon J. Chung, Sang-Ho Cha, Amanda L. Grimm, Grace Chung, Kathleen A. Gibson, Kyoung-Jin Yoon, Steven M. Parish, Chak-Sum Ho, Stephen S. Lee

**Affiliations:** 1 VMRD Inc., Pullman, WA 99163, United States of America; 2 Department of Veterinary Microbiology and Pathology, Washington State University, Pullman, WA 99163, United States of America; 3 Department of Virology, Animal and Plant Quarantine Agency, Anyang, Republic of Korea; 4 Department of Veterinary Diagnostic and Production Animal Medicine, College of Veterinary Medicine, Iowa State University, Ames, IA 50011, United States of America; 5 Department of Veterinary Clinical Sciences, College of Veterinary Medicine, Washington State University, Pullman, WA 99163, United States of America; 6 Gift of Life Michigan, Ann Arbor, MI 48108, United States of America; 7 Department of Statistics, University of Idaho, Moscow, ID 83844, United States of America; Sun Yat-Sen University, CHINA

## Abstract

**Background/Aim:**

Live attenuated vaccines confer partial protection in pigs before the appearance of neutralizing antibodies, suggesting the contribution of cell-mediated immunity (CMI). However, PRRSV-specific T-lymphocyte responses and protective mechanisms need to be further defined. To this end, the hypothesis was tested that PRRSV-specific T-lymphocytes induced by exposure to type-2 PRRSV can recognize diverse isolates.

**Methods:**

An IFN-gamma ELISpot assay was used to enumerate PRRSV-specific T-lymphocytes from PRRSV_SD23983_-infected gilts and piglets born after in utero infection against 12 serologically and genetically distinct type-1 and -2 PRRSV isolates. The IFN-gamma ELISpot assay using synthetic peptides spanning all open reading frames of PRRSV_SD23983_ was utilized to localize epitopes recognized by T-lymphocytes. Virus neutralization tests were carried out using the challenge strain (type-2 PRRSV_SD23983_) and another strain (type-2 PRRSV_VR2332_) with high genetic similarity to evaluate cross-reactivity of neutralizing antibodies in gilts after PRRSV_SD23983_ infection.

**Results:**

At 72 days post infection, T-lymphocytes from one of three PRRSV_SD23983_-infected gilts recognized all 12 diverse PRRSV isolates, while T-lymphocytes from the other two gilts recognized all but one isolate. Furthermore, five of nine 14-day-old piglets infected in utero with PRRSV_SD23983_ had broadly reactive T-lymphocytes, including one piglet that recognized all 12 isolates. Overlapping peptides encompassing all open reading frames of PRRSV_SD23983_ were used to identify ≥28 peptides with T-lymphocyte epitopes from 10 viral proteins. This included one peptide from the M protein that was recognized by T-lymphocytes from all three gilts representing two completely mismatched MHC haplotypes. In contrast to the broadly reactive T-lymphocytes, neutralizing antibody responses were specific to the infecting PRRSV_SD23983_ isolate.

**Conclusion:**

These results demonstrated that T-lymphocytes recognizing antigenically and genetically diverse isolates were induced by infection with a type 2 PRRSV strain (SD23983). If these reponses have cytotoxic or other protective functions, they may help overcome the suboptimal heterologous protection conferred by conventional vaccines.

## Introduction

Porcine reproductive and respiratory syndrome (PRRS) is one of the most economically important swine diseases worldwide. In the United States, direct economic loss due to PRRS is estimated to exceed $600 million per year [1,2]. PRRS virus (PRRSV) is an arterivirus in the *Arteriviridae* family with a single-stranded positive sense RNA genome of ~15 Kb encoding 10 open reading frames [3–5]. Rapidly and continuously increasing genetic diversity among PRRSV isolates [3,6,7] and emergence of highly pathogenic variants in different geographical regions [8–19] present a tremendous challenge to the control of PRRSV using conventional vaccines.

Both inactivated and live attenuated virus vaccines are used to aid PRRS control in swine herds, but the efficacy and/or safety of current licensed vaccines are not satisfactory [20–22]. Inactivated PRRSV vaccines induce weak neutralizing antibody responses even against homologous isolates and weaker to no response against heterologous isolates [21,23,24]. Live attenuated PRRSV vaccines contribute to clinical protection by unknown mechanisms without preventing infection, but a high probability of reversion to virulence is a major safety concern [9,25,26]. To better control PRRSV infections worldwide, it is crucial to develop a safer and more efficacious vaccine that confers protective immunity against diverse PRRSV isolates.

There have been numerous attempts to determine if neutralizing antibody provides protective immunity against PRRSV [27–33]. Passive transfer of hyperimmune serum containing high titer neutralizing antibody against PRRSV controlled viremia in pigs challenged with homologous PRRSV, but was not able to prevent viral replication in tissues [31,34]. In addition, neutralizing antibodies did not provide protection against heterologous challenge [32]. Several studies have found that immunization with live attenuated and killed PRRSV vaccines reduced clinical disease and/or viremia in pigs after PRRSV challenge, before the appearance of neutralizing antibody responses [29,30,33,35–37]. These observations, as well as the co-existence of infectious PRRSV and low titer neutralizing antibodies in the blood of infected pigs [27,28,38,39], lead to the conclusion that neutralizing antibodies alone are not potent enough to control PRRSV infection in pigs recovered from natural infections or vaccinated pigs.

Other mechanisms such as cell-mediated immunity (CMI) may contribute to control of PRRSV. Such correlates of protective immunity have not been clearly defined. Therefore, systematic approaches are needed to evaluate if CMI is more important than humoral immunity in the control of infections with diverse PRRSV isolates. A crucial step for defining the relative advantage of CMI versus humoral immunity in PRRSV-infected pigs is to determine if CMI has the potential to overcome the challenge of antigenic variation between diverse isolates. In this study, cross reactive T-lymphocyte responses against genetically and antigenically divergent PRRSV isolates were demonstrated in two pig groups of different ages using an interferon (IFN)-gamma ELISpot assay. Peptides containing T-lymphocyte epitopes were mapped to PRRSV proteins with several occurring in the M protein, one of which was recognized by T-lymphocytes from all three gilts used for mapping. These observations should help define both cytotoxic and helper T-lymphocyte epitopes that may be used for development of improved and novel PRRSV vaccines that provide protection against genetically and antigenically diverse isolates.

## Methods

### Sequencing and phylogenetic analysis of PRRSV open reading frame 5 (ORF5)

**Fig 1:**
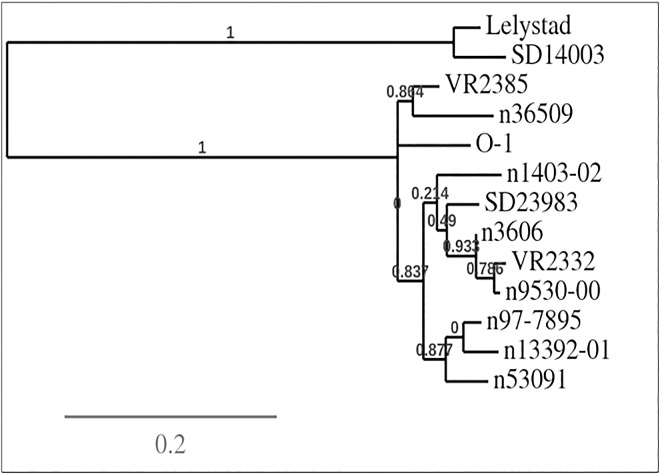
Phylogenetic analysis using open reading frame 5 sequence of 12 serologically distinct PRRSV isolates. Phylogenetic analyses were conducted using PhyML 3.0 software. Numbers in the figure represent Robinson and Foulds distance [69] measuring the topological difference between true and inferred trees.

The extraction of viral RNA from three PRRSV isolates (O-1, 3606, 14003), reverse transcription (RT) and polymerase chain reaction (PCR) amplification of the gene encoded by ORF5, and DNA sequencing were carried out as previously described [40]. Briefly, total RNA was extracted from each of virus culture supernatants using the viral RNA isolation kit from Life Technologies (Grand Island, NY, USA). The RT reaction mixture consisted of 13 μL total RNA, 1 μL of 10 mM random primer (New England Biolabs, Ipswich, MA, USA), 1 μL (50 U) of reverse transcriptase (Promega, Madison, WI, USA), 4 μL of 5× buffer (Promega), and 1 μL of 10 mM dNTPs (Life Technologies). Reverse transcription was conducted at 37°C for 30 minutes using a thermal cycler (Applied Biosystems model 2720, Life Technologies) to produce full-length cDNA. ORF5 sequences of three PRRSV strains were then PCR amplified from cDNA using 1 μL of each of 100 μM concentration primers (VR-F: TTAGCCTGTCTTTTTGCCATTCT and VR-R: TGT GGAGCCGTGCTATCAT for type-2 PRRSVs [O-1, 3606]; LV-F: ATTGCTTGTTTGTTCGCCA, LV-R: AAGGCTAGCACGAGCTTTTGT for type-1 PRRSV [SD14003]) and 9 μL of PRRSV cDNAs in a PCR mixture which contained 0.4 μL of 5U/μL high fidelity DNA polymerase (Life Technologies), 5 μL of 10X buffer, 5 μL of 50 mM MgSO_4_ (Life Technologies), 5 μL of 10 mM dNTPs, and 23.6 μL of distilled water. PCR cycles were 94°C for 5 minutes, followed by 35 cycles of 94°C for 1 minute, 53°C for 1 minute, 72°C for 1 minute, and a final extension at 72°C for 10 minutes. The PCR products were purified using the QIAquick^®^ Gel Extraction kit (QIAGEN, Hilden, Germany) and used for sequencing (Eurofins, Louisville, USA). Phylogenetic analyses were conducted of the three ORF5 sequences determined in this study coupled with 8 other sequences from GenBank (VR-2332: EF536003.1; SD23983: JX258843.1; 13392–01: EU556196.1; 53091: EU556181.1; 36509: EU556172.1; 97–7895: Ay545985.1; 1403–02: EU556164.1; 9530: EU556164.1; Lelystad: M96262.2; VR-2385: AFN88229.1) using PhyML 3.0 software (http://phylogeny.lirmm.fr) [41]. This program package consists of sequence alignment (MUSCLESetting), curation (GblocksSetting), phylogeny analysis (PhyMLSetting), and Tree Rendering (TreeDyn)Setting). These twelve PRRSV isolates were selected to stimulate T-lymphocytes based on significant genetic distances illustrated in Fig 1 and on previously reported serological distinctions [42].

### Preparation of PRRS viruses

Twelve PRRSV isolates (VR-2332 [43], SD23983 [44], 1403–02, 13392–01, 97–7895 [45], 53091, O-1, 9530–00, 3606, 36509, Lelystad [46], SD14003) were used in this study. Lelystad and SD14003 were type-1 PRRSV and all others were type-2 PRRSV. All viruses were propagated in the MARC-145 cell line [47], known to be highly permissive to PRRSV. Confluent cell monolayers prepared in 150 cm^2^ tissue culture flasks were inoculated with a 0.01–0.1 multiplicity of infection. Virus-induced cytopathic effect (CPE) reached the maximum after incubation at 37°C in Eagle’s Modified Essential Medium (EMEM; Corning, Manassas, VA, USA) supplemented with 10% fetal bovine serum (FBS; Seradigm, Radnor, PA, USA) for five to seven days, at which point culture supernatants were combined and centrifuged at 4,000 × *g* for 10 minutes at 4°C to eliminate cell debris.

The titer of PRRSV in each cell culture supernatant was determined using a microtitration infectivity assay and the median tissue culture infective dose (TCID_50_) calculated according to the method of Reed and Muench [48]. Briefly, confluent monolayers of MARC-145 cells prepared in 96-well plates (Corning) were inoculated with 10-fold serial dilutions of PRRSV prepared in EMEM with 10% FBS. The development of CPE in the cells was monitored daily for seven days and the results were used to calculate TCID_50_/mL.

### PRRSV-infected pigs

Three 12-month-old gilts (numbered 1–3) from the same dam were determined to be negative for porcine circovirus, porcine epidemic diarrhea virus, and PRRSV by realtime PCR and immunofluorescent antibody assay. They were then intranasally inoculated with 1.6 x 10^4^ TCID_50_ of PRRSV_SD23983_ and subsequently maintained in isolation rooms at Washington State University for five months. All three gilts demonstrated clinical disease in the form of mild nasal discharge but recovered clinically within two weeks after PRRSV infection. Of three gilts, gilt-2 had positive viremia by realtime PCR at 2 (23.1 Ct), 4 (23.5 Ct), 6 (32.2 Ct), 8 (35.3 Ct) and 12 (35.5 Ct) dpi, and negative viremia (≥37.0 Ct) after 12 dpi. Gilt-3 had weak positive viremia (36.0 Ct) at 4 dpi only and negative viremia at later dpi. Gilt-1 had no detectable viremia (≥37.0 Ct) at all tested dpi although appearance of PRRSV-specific antibodies after intranasal inoculation of PRRSV SD23983 clearly demonstrates the infection. These gilts were the source of peripheral blood mononuclear cells (PBMC) collected at 30–33, 44, 58 and 72 days post-infection (dpi) for *in vitro* stimulation with PRRSV or peptides. Gilt 1 was artificially inseminated with semen of a PRRSV-negative boar and then infected with the virus at approximately 98 days of gestation. Nine piglets (numbered P13-P21) were born to gilt 1 at approximately 117 days of gestation and confirmed PRRSV-infected at birth prior to ingestion of colostrum, indicating in utero infection. PBMC were also collected from the nine piglets at 14 days of age (33 dpi). The animal use protocols for these animal studies were approved by the Institutional Animal Care and Use Committees at Washington State University. Proper sedation and anesthesia were used to minimize potential pain in sampling procedure from the gilts used in this study.

### MHC class I and II typing of gilts and piglets

Gilts 1–3 and the nine piglets used in this study were genotyped for three swine leukocyte antigen (SLA) class I genes (SLA-1, -2, -3) and three class II genes (DRB1, DQB1, DQA) using the low resolution PCR-SSP (polymerase chain reaction with sequence-specific primers) typing panels as previously described [49,50]. Low resolution SLA class I and class II haplotypes were deduced based on published (15713212, 16305679, 18760302, 19317739) and unpublished (http://www.jimmunol.org/cgi/content/meetingabstract/186/1_MeetingAbstracts/170.1) haplotypes identified in outbred commercial pigs.

### Preparation of peripheral blood mononuclear cells and storage

Swine PBMC from fresh venous blood collected in 5 mM EDTA (final concentration) were isolated by centrifugation on a discontinuous gradient using Lymphoprep™ (STEMCELL™ Technologies, Vancouver, Canada) as previously described [51]. Briefly, blood with 5 mM EDTA was diluted two-fold in 10mM phosphate-buffered saline (PBS, pH 7.4) containing 20% anti-coagulant citrate dextrose (ACD) and 2% FBS. Thirty-five milliliters of the diluted blood was added to a SepMate™-50 tube (STEMCELL Technologies) containing 15 mL of Lymphoprep™ and the tubes were centrifuged at 1,100 × *g* for 10 minutes. The buffy coat cells within the supernatant were collected and rinsed with PBS containing 20% ACD and 2% FBS until platelets were removed. Isolated PBMCs were counted, resuspended in a freezing medium containing 10% dimethyl sulfoxide (J.T. Baker, Center Valley, PA, USA) and 90% FBS, and stored in liquid nitrogen until use.

### IFN-gamma enzyme-linked immunospot (ELISpot) assay

The IFN-gamma ELISpot assay used to define PRRSV-specific T-lymphocyte responses was performed using a commercial product (Mabtech, Cincinnati, OH, USA) as per manufacturer’s instructions. In brief, ELISpot plates were coated with anti-IFN-gamma antibody (1 ng/well) diluted in sterile 10 mM PBS (pH 7.4) by incubating at 4°C overnight. Wells were washed five times with PBS and blocked/equilibrated with RPMI-1640 media (Life Technology, Grand Island, NY, USA) containing 10% FBS for two hours at ambient temperature. PBMC (10^5^ to 10^6^ cells) in 100 μL of RPMI-1640 media supplemented with 10% FBS were added to each well of ELISpot plates. Each PRRSV antigen (e.g. live PRRSV at 10^5^ TCID_50_, inactivated PRRSV or peptides at one to four μM) or mitogen (PHA 0.5 μg) suspended in 100 μL of RPMI-1640 media was then added to each well. The plates were incubated at 37°C for approximately 20 hours in a water-jacketed incubator conditioned with 5% CO_2_. To detect the spots associated with IFN-gamma secretion from antigen-specific T-lymphocytes, the plates were washed five times with PBS and biotinylated anti-IFN-gamma antibody (0.05 μg/100 μL/well) was added. Following two hours of incubation at ambient temperature, the plates were washed and then incubated with 100 μL of 1000-fold diluted streptoavidin-HRP (Mabtech) per well for one hour at ambient temperature. The plates were again washed five times with PBS and, following the addition of 100 μL of substrate solution, color spots were developed for approximately 90 seconds. The colorimetric reaction was stopped by rinsing with distilled water, and the plates were dried in the dark until spot forming units (SFU) were analyzed by an ELISpot reader (Autoimmun Diagnostika GmbH, Strasberg, Germany). The mean SFU/10^6^ PBMC and standard error (SE) were calculated for the three non-stimulated negative control wells. For the test wells (two per test unless otherwise indicated), the mean was calculated and the negative control mean subtracted to determine mean antigen-specific SFU (representative data in S1 Fig). Those tests with a mean antigen-specific SFU higher than the mean SFU in non-stimulated wells plus five times the SE (≥99% confidence interval) were considered significant positive results.

### T-lymphocyte epitope mapping

Peptides consisting of 20 amino acids with 11 amino acid overlaps were synthesized for all structural and nonstructural proteins of PRRSV_SD23983_ by a commercial manufacturer (Mimotope Pty Ltd., Clayton, Australia). These synthetic peptides were used in ELISpot assay to define the location of epitopes recognized by T-lymphocytes from PRRSV-infected gilts.The initial round of screening was with pools containing 33–54 peptides per pool (~1 μM of each peptide) and those that were positive were tested in the second round with smaller pools containing two to four peptides (4 μM of each). The third round of screening tested each peptide individually from positive pools identified in the second round (4 μM of each). All individual peptides inducing a significant T-lymphocyte response in the third round were retested with four replicates of each peptide.

### Fluorescent focusing neutralization (FFN) assay

The PRRSV neutralizing antibody response in serum was measured by FFN assay as previously described [52]. Briefly, two-fold serial dilutions (1:4 to 1:512) of heat-inactivated serum samples were prepared in 96-well plates using Minimum Essential Medium (MEM, GIBCO^®^ brand; Life Technology, Grand Island, NY, USA) supplemented with 10% FBS. An equal volume (50 μL) of PRRSV (VR-2332 or SD23983 strain) at a concentration of 2 × 10^3^ FFU_50_ per ml was added to each sample, incubated for one hour at 37°C, and transferred to a 96-well plate containing confluent MARC-145 cells. After 24 hours, the plates were washed, fixed in ice-cold 80% acetone (Fisherbrand^®^; Fisher Scientific, Hanover Park, IL, USA), and stained with fluorescein isothiocyanate-conjugated anti-PRRSV nucleocapsid monoclonal antibody SDOW17 (Rural Technologies, Brookings, SD, USA) diluted 1:100 in PBS. The neutralizing activity of each serum (i.e., FFN titer) was reported as the reciprocal of the highest dilution that caused a 90% or greater reduction in the number of fluorescent foci.

## Results

### T-lymphocytes from pigs infected with type-2 PRRSV_SD23983_ recognized highly diverse type-1 and -2 PRRSV isolates

To define T-lymphocyte responses reacting against diverse PRRSV isolates, PBMCs collected from three one-year-old gilts at 44 and 72 days following infection with type-2 PRRSV_SD23983_ were evaluated by IFN-gamma ELISpot assay using 12 diverse PRRSV isolates as antigen. Gilts 1 and 3 had identical MHC haplotypes (Class I = Lr-32.0/35.0, Class II = Lr-0.1/0.12) and had broadly cross-reactive PRRSV-specific T-lymphocyte responses at 44 dpi, recognizing 11 of 12 heterologous PRRSV isolates significantly high (>5 SE) (Table 1). Notably, one of the recognized isolates included the type-1 PRRSV_Lelystad_, which has more than 40% difference in GP5 and complete ORF sequences from major type-2 PRRSV isolates, including the type-2 PRRSV_SD2398_ inoculum virus as well as PRRSV_VR2332_ (Fig 1). Gilt 2 had a different MHC haplotype (Class I = Lr-4.0/39.0, Class II = Lr-0.2/0.23) and demostrated significantly high (>5 SE) T-lymphocyte responses against 10 of 12 strains at 44 dpi. By 72 dpi T-lymphocytes from gilt 3 had significantly high (>5 SE) recognition of all 12 PRRSV isolates and those from gilts 1 and 2 recognized 11 of 12 isolates (Table 1).

**Table 1:** T-lymphocyte responses from one-year-old gilts infected with PRRSV_SD23983_ at 44 and 72 dpi in an IFN-gamma ELISpot assay against various type-1 and type-2 PRRSV isolates.

PRRSV type: strain	44 dpi PBMC			72 dpi PBMC		
	Gilt 1	Gilt 2	Gilt 3	Gilt 1	Gilt 2	Gilt 3
Unstimulated: mean SFU (5 x SE)	2 (5)	6.5 (2.5)	15 (30)	4 (0)	28 (5)	30 (10)
1: PRRSV_LV_	43+*	11.5+	97+	73+	46+	>262+
1: PRRSV_14003_	6	6	7	4	24	89+
2: PRRSV_VR2332_	148+	61+	242+	>262+	>262+	>262+
2: PRRSV_13392_	199+	71+	262+	>262+	>262+	>262+
2: PRRSV_53091_	136+	72+	218+	>262+	>262+	>262+
2: PRRSV_36509_	>262+	69+	218+	>262+	>262+	>262+
2: PRRSV_97-7895_	>262+	61+	230+	>262+	>262+	>262+
2: PRRSV_O-1_	>262+	59+	>262+	>262+	>262+	>262+
2:PRRSV_1403-02_	>262+	61+	238+	>262+	>262+	>262+
2: PRRSV_9530_	>262+	58+	210+	>262+	>262+	>262+
2: PRRSV_3606-98_	165+	4	139+	>262+	>262+	>262+
2: PRRSV_SD23983_	259+	52+	226+	>262+	>262+	>262+

*Numbers are mean SFU/10^6^ PBMC minus the mean of the appropriate unstimulated control; those adjusted SFU means that were greater than the unstimulated control SFU mean plus 5 times the SE (standard error) were scored as positive and marked with a + in the table (for gilt 1 at 44 dpi those test SFU means >7 were positive; for gilt 2, >9; for gilt 3, >45 and so on). SFU means with >262+ indicate that the SFU was higher than the upper count limit for ELISpot reader.

T-lymphocytes from nine 14-day old piglets born to gilt 1 after in utero infection with PRRSV_SD23983_ were evaluated by IFN-gamma ELISpot assay using the 12 described PRRSV isolates. The infection period totaled 33 days (including 19 days in utero and 14 days after birth) to allow stimulation of PRRSV_SD23983_-specifc T-lymphocytes in all piglets. All nine had significant (>5 SE) T-lymphocyte responses against two or more of the heterologous PRRSV isolates tested (Table 2). In particular, piglet 13 had T-lymphocytes recognizing all 12 PRRSV isolates including the two type-1 PRRSV isolates.

**Table 2:** T-lymphocyte responses from 14-day-old piglets infected in utero with PRRSV_SD23983_ in an IFN-gamma ELISpot assay against phytohemagglutin (PHA) and various type-1 and type-2 PRRSV isolates.

	Pig number and SLA genotype								
PHA or PRRSV type: isolate	P13	P14	P15	P16	P17	P18	P19	P20	P21
	Lr49.29+Lr35.12	Lr49.29+Lr32.1	Lr4.2+Lr32.1	Lr4.2+Lr32.1	Lr4.2+Lr35.12	Lr4.2+Lr32.1	Lr49.2+Lr32.1	Lr4.2+Lr32.1	Lr4.2+Lr35.12
PHA	<353	337 (29)	<353	149 (6)	<353	220 (26)	<353	205 (87)	<353
Unstimulated: mean SFU (5 x SE)	6.5 (7.5)	11 (20)	6.5 (2.5)	10.5 (12.5)	21 (45)	1 (5)	9.5 (17.5)	2 (5)	7 (0)
1: PRRSV_LV_	63+*	4	24+	0	3	3	3	0	13+
1: PRRSV_14003_	40+	7	3	0	0	1	15	0	1
2: PRRSV_VR2332_	320+	45+	3	0	30	15+	38+	23+	60+
2: PRRSV_13392_	181+	28	20+	15	32	7+	86+	28+	30+
2: PRRSV_53091_	231+	30	17+	13	71+	9+	37+	18+	30+
2: PRRSV_36509_	235+	30	23+	4	27	8+	25	0	69+
2: PRRSV_97-7895_	353+	27	27+	34+	69+	21+	51+	0	71+
2: PRRSV_O-1_	285+	25	44+	11	47	18+	46+	0	104+
2:PRRSV_1403-02_	235+	23	50+	29+	38	7+	67+	76+	68+
2: PRRSV_9530_	276+	41+	27+	6	51	6	43+	0	66+
2: PRRSV_3606-98_	214+	25	27+	2	30	11+	43+	0	76+
2: PRRSV_SD23983_	329+	51+	30+	9	60	17+	132+	5	73+

* Numbers are mean SFU/10^6^ PBMC minus the mean of the appropriate unstimulated control; those adjusted SFU means that were greater than the unstimulated control SFU mean plus 5 times the SE (standard error) were scored as positive and marked with a + in the table (for P13 those test SFU means >14 were positive; for P14, >31; for P15, >9 and so on).

### T-lymphocytes from three gilts infected with PRRSV_SD23983_ at one year of age recognized several epitopes in various viral proteins

PRRSV epitopes were initially mapped with high stringency IFN-gamma ELISpot assays using 20 peptide pools (33 to 54 overlapping peptides per pool) spanning all PRRSV_SD23983_ ORFs. In the first round of epitope mapping, PBMC collected at 30 or 33 dpi had significant T-lymphocyte responses against four to 14 of the 20 peptide pools, depending on the source animal (Table 3). This suggested that T-lymphocyte responses to multiple epitopes in several proteins contributed to recognition of diverse PRRSV isolates. In general, the number of peptide pools recognized by T-lymphocytes from the three gilts at 44 or 58 dpi was similar to the findings at 30–33 dpi (Table 3).

**Table 3:** Mapping of T-lymphocyte epitopes on PRRSV proteins using overlapping peptide pools encompassing all open reading frames of PRRSV_SD23983_.

Peptide pool	Gilt 1 PBMC			Gilt 2 PBMC			Gilt 3 PBMC		
	30dpi	44dpi	58dpi	33dpi	44dpi	58dpi	33dpi	44dpi	58dpi
Unstimulated: mean SFU (5 x SE)	19 (15)	60.5 (52.5)	24.5 (2.5)	37 (10)	3 (10)	4 (5)	26.5 (22.5)	6 (5)	5.5 (7.5)
nsp1	129+*	183+	141+	26	15	24+	29	18+	9
nsp2-1	49+	92	56+	2	8	13+	13	3	1
nsp2-2	133+	245+	179+	18	0	8	34	13+	9
nsp2-3	31	45	30+	19	0.5	0	9	0	0
nsp3,4	49+	72	47+	115+	3	3	20	1	0
nsp5,6,7	76+	75	73+	26	6	11+	16	6	2
nsp8,9	29	22	27	55+	4	8	11	5	3
nsp9,10	35+	48	30+	10	1	4	14	1	0
nsp10,11	20	44	21	9	1	3	23	0	0
nsp12, 2TF	20	18	20	4	0	2	11	9	0
GP2a and b, ORF5a	143+	141+	74+	17	7	27+	28	46+	11
GP3, GP4	212+	226+	180+	59+	25+	47+	60+	74+	19+
GP5, M, N	333+	>345+	308+	56+	30+	65+	88+	155+	80+
M	218+	180+	134+	26	35+	93+	61+	129+	55+
E (GP2b)	14	13	16	6	1	5	4	7	4
GP3	42+	29	28+	27	17+	46+	17	8	3
GP4	194+	163+	139+	38	24+	42+	33	65+	26+
GP5	200+	263+	214+	23	13	25+	65+	75+	34+
ORF5a	11	19	14	25	1	0	22	6	2
N	22	NA	NA	3	4	7	7.5	14+	6
Inactivated PRRSV_SD23983_	182+	204+	177+	27	0	3	51+	1	0

* Numbers are mean IFN-gamma ELISpot assay SFU/10^6^ PBMC minus the mean of the appropriate unstimulated control; those adjusted SFU means that were greater than the unstimulated control SFU mean plus 5 times the SE were scored as positive and marked with a + in the table (for gilt 1 at 30 dpi those test SFU means >34 were positive; for gilt 1 at 44 dpi, >113; for gilt 1 at 58 dpi, >27 and so on). SFU means with > indicate that the SFU was higher than the upper count limit for ELISpot reader.

Second and third round T-lymphocyte epitope mapping identified a total of 28 peptides recognized by T-lymphocytes from gilt 1 which included four overlapping regions, resulting in mapping of epitopes on ≥24 peptides (Table 4). The viral proteins containing these peptides with epitopes were nsp1 alpha (five peptides, five epitopes), nsp2 (five peptides, ≥five epitopes), GP2a (two peptides, ≥two epitopes), GP2b (one peptide, ≥one epitope), GP4 (six peptides, ≥five epitopes), GP5 (three peptides, ≥three epitopes) and M (six peptides, ≥three epitopes). Gilt 2 T-lymphocytes recognized six peptides including two overlapping regions (≥four epitopes) in viral proteins nsp4 (two peptides, ≥one epitope), GP3 (one peptide, ≥one epitope), GP4 (one peptide, ≥one epitope) and M (two peptides, ≥one epitopes). Gilt 3 T-lymphocytes recognized seven peptides including two overlapping regions (≥five epitopes) in viral proteins GP5 (one peptide, ≥one epitope), M (five peptides, ≥three epitopes) and N (one peptide, ≥one epitope). Of these seven peptides, six were also recognized by T-lymphocytes from gilt 1 with the same MHC class I and II haplotypes. It is noteworthy that one peptide (P579 TWKFITSRCRLCLLGRKYIL) in the M protein was recognized by T-lymphocytes from all three gilts representing two distinct MHC class I and II haplotypes.

**Table 4:** Peptides containing epitopes recognized by T-lymphocytes from three gilts infected with PRRSV_SD23983_.

Pig ID (dpi)	Peptide number and sequence	Protein	Mean SFU (5 x SE)
Gilt 1 (30)	Unstimulated		2.2 (6.5)
	P2 CTPNARVFMAEGQVYCTRCL	nsp1alpha	12+*
	P10 FPIARMTSGNLNFQQRMVRV	nsp1alpha	12+
	P13 GQLTPAVLKALQVYERGCRW	nsp1alpha	21+
	P15 RWYPIVGPVPGVAVYANSLH	nsp1alpha	148+
	P18 GATHVLTNLPLPQRPKPEDF	nsp1alpha	25+
	P53 ATDEDLVNAIQILRLPAALD	nsp2	14+
	P94 APRRKVGTNCGSPISLGDNI	nsp2	48+
	P121 SAYQAFRTLDGRLKFLPKMI	nsp2	16+
	P132 PDESTSAPPTGTGGAGSFTD	nsp2	22+
	P136 TIKRKAEGLFDRLSRQVFNL	nsp2	25+
	P470 TKHPLGMFWHHKVSTLIDEM	GP2a	18+
	P478 ASRLPMLHNLRMTGSNVTIV	GP2a	39+
	P493 VVFCIRLVCSAILRTRPAIH	E (GP2b)	12+
	P528 VLQDISCLRHRNSAPEALRK	GP4	42+
	P530 RKIPQCRTAIGTPVYITITA	GP4	23+
	P532 TANVTDENYLHSSDLLMLSS	GP4	30+
	P533 LHSSDLLMLSSCLFYASEMS	GP4	30+
	P538 QHVREFTQRSLMVDHVRLLH	GP4	16+
	P540 LHFMTPETMRWATVLACLFA	GP4	22+
	P550 LTHIVSYGALTTSHFLDTIA	GP5	25+
	P555 AALTCFVIRFVKNCMSWRYS	GP5	17+
	P558 LLDTKGRLYRWRSPVIIEKR	GP5	96+
	P571 LLAFSITYTPVMIYALKVSR	M	24+
	P572 PVMIYALKVSRGRLLGLLHL	M	65+
	P573 SRGRLLGLLHLLIFLNCAFT	M	21+
	P578 LWGVYSAIETWKFITSRCRL	M	23+
	P579 TWKFITSRCRLCLLGRKYIL	M	24+
	P586 LVLGGRKAVKQGVVNLVKYA	M	65+
	Total 28 peptides: ≤24 epitopes		
Gilt 2 (33)	Unstimulated		5.3 (8.5)
	P221 PLGDVKVGSHIIKDIGEVPS	nsp4	34+
	P222 HIIKDIGEVPSDLCALLAAK	nsp4	24+
	P521 LGIATRPLRRFAKSLSAVRR	GP3	15+
	P538 QHVREFTQRSLMVDHVRLLH	GP4	24+
	P579 TWKFITSRCRLCLLGRKYIL	M	35+
	P580 RLCLLGRKYILAPAHHVESA	M	20+
	Total 6 peptides: ≤4 epitopes		
Gilt 3 (33)	Unstimulated		3.5 (7.5)
	P558 LLDTKGRLYRWRSPVIIEKR	GP5	25+
	P571 LLAFSITYTPVMIYALKVSR	M	22+
	P572 PVMIYALKVSRGRLLGLLHL	M	23+
	P578 LWGVYSAIETWKFITSRCRL	M	17+
	P579 TWKFITSRCRLCLLGRKYIL	M	10+
	P586 LVLGGRKAVKQGVVNLVKYA	M	37+
	P589 KRKKGDGQPVNQLCQMLGKI	N	15+
	Total 7 peptides: ≤5 epitopes		

* Numbers are mean IFN-gamma ELISpot assay SFU/10^6^ PBMC minus the mean of the appropriate unstimulated control; those adjusted SFU means that were greater than the unstimulated control SFU mean plus 5 times the SE were scored as positive and marked with a + in the table: for gilts 1, 2 and 3 were >8.7, >13.5 and >11, respectively.

### Neutralizing antibody responses were weak and isolate-specific while T-lymphocytes recognized diverse isolates

To define neutralizing antibody responses, virus neutralizing antibody titers were determined by FFN assay using two type-2 PRRSV, the inoculum PRRSV_SD23983_ as well as PRRSV_VR2332_, which are closely related according to phylogenetic analysis (Fig 1). All three gilts had weak to moderate neutralizing antibody titers against the homologous PRRSV_SD23983_, ranging between 1:4 and 1:32 at 30–72 dpi (Table 5). However, there was no detectable level of neutralizing antibodies against the heterologous but closely related PRRSV_VR2332_ in any of the serum samples from various dpi time points tested (Table 5).

**Table 5:** Neutralizing antibody responses in three gilts after intranasal challenge with PRRSV_SD23983_ and in piglets at 14-days of birth to gilt 1 exposed to PRRSV_SD23983_ at 98 days of gestation.

Pig identification	dpi	Neutralizing antibody titer against homologous isolate	Neutralizing antibody titer against VR2332 isolate
Gilt 1	30	1:4*	<1:4
Gilt 1	58	1:16	<1:4
Gilt 1	72	1:8	<1:4
Piglet 13	32**	1:16	<1:4
Piglet 14	32	1:16	<1:4
Piglet 15	32	1:32	<1:4
Piglet 16	32	1:16	<1:4
Piglet 17	32	1:32	<1:4
Piglet 18	32	1:8	<1:4
Piglet 19	32	1:16	<1:4
Piglet 20	32	1:16	<1:4
Piglet 21	32	1:16	<1:4
Gilt 2	30	1:32	<1:4
Gilt 2	58	1:32	<1:4
Gilt 2	72	1:32	<1:4
Gilt 3	30	1:16	<1:4
Gilt 3	58	1:32	<1:4
Gilt 3	72	1:32	<1:4

*Neutralizing antibody titer is the last serum dilution with detectable neutralizing activity.

**32 dpi for piglets born to gilt 1 includes 18 days gestation following infection of gilt plus 14 days after birth, at which point the serum sample was obtained.

Five of nine piglets had PRRSV-specific T-lymphocyte responses reacting against diverse type 2 PRRSV isolates (≥9 of 12 isolates). Of two type-1 isolates, piglet 13 significantly (>5SE) recognized both, while piglets 15 and 21 each recognized one. In contrast, neutralizing antibody responses in all nine piglets were weak against the homologous PRRSV_SD23983_ isolate with titers ranging between 1:8 and 1:32, and there was no neutralization of slightly heterologous PRRSV_VR2332_ (Table 5). Neutralization results were similar to serum from the dam (gilt 1), with isolate-specific neutralization (Table 5).

## Discussion

PRRSV infects porcine macrophages using CD163 and CD169 as receptors [53–56]. This virus then persists in tissue macrophages and modulates the immune system, resulting in weak and/or delayed adaptive and innate immune responses [51,57,58]. Neutralizing antibody responses are weak and inefficient in preventing PRRSV infection of target cells in tissues even in a homologous challenge model [31]. Therefore, CMI has been frequently discussed as a protective mechanism against PRRSV infection [58–64] [65] [66]. Studies to date have demonstrated the existence of T-lymphocyte epitopes in several PRRSV proteins including both structural (GP2, GP3, GP5, M, N) and non-structural proteins (nsp1, nsp2, nsp5, nsp7, nsp9 and nsp10) [58–64]. PRRSV-specific T-lymphocytes in PBMC from infected pigs appeared at 14 dpi and remained longer than 50 days when evaluated in IFN-gamma ELISpot assay using the mixture of PRRSV_MN30100_ and recombinant N protein as the stimulation antigen [65]. Furthermore, PRRSV-specific T-lymphocyte responses were highly variable between different age groups, potentially related to infection strains and virus titer [66]. To date, no systematic evaluation of the cross-recognition potential of PRRSV-specific T-lymphocyte responses to heterologous PRRSV isolates has been performed. If T-lymphocyte responses recognize multiple epitopes in diverse PRRSV isolates and have a protective function, they may overcome the challenges presented by antigenic variation among PRRSV isolates and weak, isolate-specific neutralizing antibody responses.

The objective of this study was to define broadly cross-reactive PRRSV-specific T-lymphocyte responses induced in gilts and piglets after infection with a type-2 PRRSV and map epitope-containing sequence within T-lymphocyte-recognized viral proteins. T-lymphocytes from both age groups recognized multiple diverse PRRSV isolates. In the three 12-month-old gilts, intranasal infection with PRRSV_SD23983_ induced T-lymphocyte responses that reacted against 10 type-2 isolates and one or both of the two highly divergent type-1 isolates when evaluated by 72 dpi. The recognition of all 12 PRRSV isolates including two type-1 isolates by gilt 3 T-lymphocytes was unexpected considering the antigenic and genetic differences between inoculum PRRSV_SD23983_ and tested divergent isolates. T-lymphocytes from all nine 14-day-old piglets infected in utero recognized at least two of the 12 PRRSV isolates tested. Importantly, T-lymphocytes from one of the nine piglets recognized all 12 PRRSV isolates and another recognized 11 isolates. This finding could be reinforced through further studies with more diverse PRRSV isolates including high pathogenic strains as well as PBMC at various dpi time points.

The broadly cross-reactive T-lymphocyte responses observed in this study were in direct contrast to the weak and isolate-specific neutralizing antibody responses to the homologous PRRSV_SD23983_ in gilts when tested at various dpi time points. This strongly supports the effort to define T-lymphocyte responses further with regard to protective mechanisms. Porcine T-lymphocytes that could contribute to protective responses include CD4^+^ T-lymphocytes, CD8^+^ T-lymphocytes and memory T-lymphocytes with the double positive CD4^+^CD8^+^ phenotype. To this end, a companion study using T-lymphocytes from the three gilts in this study demonstrated cytotoxic responses by both CD8^+^ and CD4^+^ T-lymphocytes [67]. Additional large scale studies using pigs with systematically defined MHC haplotypes are also indicated to investigate the association between MHC haplotypes and PRRSV-specific IFN-gamma-secreting T-lymphocyte responses, as this limited analysis of gilts and congenitally infected piglets is not definitive.

The epitope mapping results in this study identified peptides containing T-lymphocyte epitopes in multiple proteins including nsp4, in which no other T-lymphocyte epitopes have been reported to date. T-lymphocyte epitopes have been previously described in PRRSV nsp1, nsp2, GP2, GP4, GP5, M and N proteins [58–64], however in this study the exact locations for 23 of 33 epitope-containing peptides identified in these proteins were novel. Locations of ten peptides including seven M peptides, two GP5 peptides, and one nsp2 peptide were similar to those previously reported. M protein had several epitopes recognized by two of three gilts tested and, in particular, one epitope-containing peptide (P579) that was recognized by T-lymphocytes from all three. It will be interesting to determine if either cytotoxic T-lymphocytes (CTL) and/or helper T-lymphocytes recognize this peptide and whether they potentially recognize the same epitope. In addition, the same repertoire of epitope-containing peptides from the M protein were recognized by two gilts (gilt 1 and gilt 3) with identical MHC class-I and -II haplotypes. Despite differing MHC class-I and -II haplotypes, T-lymphocytes in gilt 1 and 2 recognized a common epitope-containing peptide (P538) in GP4. Identification of M protein sequences recognized by T-lymphocytes in several previous reports [58,59,62,68] as well as in the current study suggest that M protein is epitope-rich region suitable for inducing T-lymphocytes recognizing diverse PRRSV isolates, particularly since PBMCs have originated from genetically diverse pigs infected with various PRRSV genotypes.

Four PRRSV non-structural proteins (nsp5, nsp7, nsp9 and nsp10) reported to contain T-lymphocyte epitopes in previous studies [58,63,64] had no epitopes identified in this study. This may have been due to the use of only three gilts for epitope mapping and/or the use of limited dpi time points. Additional studies are planned to undertake epitope mapping and functional analysis of T-lymphocytes through virus suppression and cytotoxic assays using PBMC collected at various dpi from pigs infected with type-1 and type-2 PRRSV strains in order to extend the current findings. In addition, inclusion of high pathogenic PRRSV strains in diverse PRRSV panel as T-lymphocyte stimulation antigen in ELISpot assay may be valuable. Thorough epitope mapping will be crucial in the development of the next generation of PRRSV vaccines, particularly those epitopes that induce CTL and helper T-lymphocytes for both CTL and B-lymphocyte responses. Epitopes recognized by helper T-lymphocytes can be used as an adjuvant for either conventional or novel PRRSV vaccines to boost PRRSV-specific T- or B-lymphocyte responses, and PRRSV-specific CTL epitopes could be included to improve protective vaccine efficacy.

## Supporting Information

S1 Fig PRRSV-specific IFN-gamma-secreting cells in PBMC from a PRRSV-infected and recovered gilt. (DOCX)
