# Supplementary material for: Recognition of Highly Diverse Type-1 and -2 Porcine Reproductive and Respiratory Syndrome Viruses (PRRSVs) by T-Lymphocytes Induced in Pigs after Experimental Infection with a Type-2 PRRSV Strain
Source: PLoS One. 2016 Oct 31;11(10):e0165450. doi: 10.1371/journal.pone.0165450 (PMC5087905; doi:10.1371/journal.pone.0165450)
Supplement: S1 Fig — (DOCX) [file pone.0165450.s001.docx]

S1 Fig. PRRSV-specific IFN-gamma-secreting cells in PBMC from a PRRSV-infected and recovered gilt

The figure is representative PBMC wells added with PRRSV or overlapping peptide pools for different open reading frames of PRRSV. Spot form unit (SFU) of each treatment well was determined as summarized below.

| 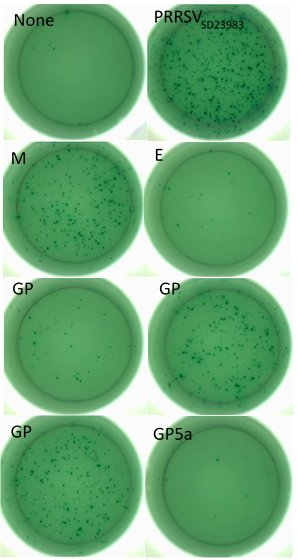 | \| Stimulation antigen \| SFU/million PBMC \| \| --- \| --- \| \| Unstimulated \| 19 \| \| M \| 237 \| \| E \| 33 \| \| GP3 \| 61 \| \| GP4 \| 213 \| \| GP5 \| 219 \| \| ORF5a \| 30 \| \| PRRSV_SD23983_ \| 359 \| |
| --- | --- | --- | --- | --- | --- | --- | --- | --- | --- | --- | --- | --- | --- | --- | --- | --- | --- | --- | --- |
